# Increased proportion of follicular helper T cells is associated with B cell activation and disease severity in IgA nephropathy

**DOI:** 10.3389/fimmu.2022.901465

**Published:** 2022-08-02

**Authors:** Wanshan Du, Cai-Yue Gao, Xing You, Liang Li, Zhi-Bin Zhao, Mengting Fang, Zhiming Ye, Meijun Si, Zhe-Xiong Lian, Xueqing Yu

**Affiliations:** ^1^School of Medicine, South China University of Technology, Guangzhou, China; ^2^Department of Nephrology, Guangdong Provincial People’s Hospital, Guangdong Academy of Medical Sciences, Guangzhou, China; ^3^Medical Research Center, Guangdong Provincial People’s Hospital, Guangdong Academy of Medical Sciences, Guangzhou, China; ^4^School of Biomedical Sciences and Engineering, South China University of Technology, Guangzhou, China; ^5^Guangdong-Hong Kong Joint Laboratory on Immunological and Genetic Kidney Diseases, Guangzhou, China

**Keywords:** IgA nephropathy, single-cell RNA sequencing, follicular helper T cell, B cell, cell-cell interaction

## Abstract

IgA nephropathy (IgAN) is the most common primary glomerulonephritis, characterized by glomerular deposition of IgA immune complexes, mainly produced by B cells under the regulation of CD4^+^T cells. However, the alterations of specific CD4^+^T cell subsets and the mechanism of B cells activation in IgAN remain unclear. Therefore, we aimed to investigate the landscape characteristics and role of CD4^+^T cells in the progression of IgAN. We identified that the proportion of Th2, Th17 and Tfh (follicular helper T) cells in patients with IgAN was significantly higher than that of healthy controls (*P* < 0.05). Single-cell RNA sequencing of peripheral blood mononuclear cells (PBMCs) showed that Th cells and B cells in patients with IgAN were more activated. Correspondingly, multiplex immunohistochemistry staining of renal biopsy showed increased infiltration of CD4^+^T and B cells in the kidneys of patients with IgAN. The degree of infiltration was positively correlated with the degree of renal damage. Interestingly, the proportion of Tfh cells in peripheral blood was positively correlated with the severity of proteinuria. Moreover, the proximity position of Tfh cells and B cells suggested that cell-cell interactions between Tfh and B cells were happening *in situ*. Intercellular communication analysis also showed enhanced interaction between Tfh cells and B cells in IgAN. Our findings suggested that Tfh cells of patients possibly contributed to the progression of IgAN by activating B cells *via* cell-cell interactions and TNFSF14-TNFRSF14 may be an underlying signaling pathway.

## Introduction

IgA nephropathy (IgAN) is the most common primary glomerulonephritis (1–3), accounting for about 40-50% of primary glomerular diseases in China, with 30–40% of patients developing end­stage renal disease (ESRD) within 20 years of onset (1–3). IgAN has been identified as an autoimmune disease characterized by the deposition of pathogenic IgA1 immune complexes in the glomerular mesangium, which then activate mesangial cells and the immune system to induce renal injury (1).

Upon recognition of antigen-major histocompatibility complex molecules and proper co-stimulation, naive CD4^+^T cells exit from quiescence to undergo clonal expansion and differentiate into specific effector Th cells, consisting of Th1, Th2, Th17, follicular helper T cells (Tfh) and regulatory T cells (Tregs) (4, 5). Th cell polarization imbalance plays a significant role in inflammatory and autoimmune diseases (5–7). As an Immunoglobulin-associated disease, the role of CD4^+^T helper (CD4^+^Th) cells, which are critical for B cells differentiation and maturation, in IgAN are not fully understood yet.

In most contexts, the development of germinal center B (GC B) cells and plasma cells (PCs) depends on Tfh cells (8–10). Tfh cells provide IL-21 and CD40L signals for B cell proliferation and differentiation towards both GC and extrafollicular fates (11). Failure of the appropriate signals such as cytokines or ligand-receptor activation occurring during B cell differentiation can contribute to the development of autoimmune diseases. The tumor necrosis factor superfamily of ligand (TNFSF) and its receptor (TNFRSF) provide vital communication signals in immune cells during development, maintaining organ homeostasis and initiating tissue responses. Among them, LIGHT (TNFSF14) activates the HVEM-TRAF2/3 E3 ligase pathway to promote cell survival and differentiation (12–14). It was reported that dysregulated LIGHT expression on T cells promoted intestinal inflammation and contributed to elevated serum IgA levels and renal IgA deposition in mice (15). These observations suggest that dysregulated TNFSF costimulatory signals in CD4^+^T cells and B cells might be involved in the development of IgAN. Interestingly, we found the transcriptional levels of TNFSF14 and TNFSFR14 were positively correlated with that of ICOS and MS4A1 respectively through analyzing a published renal transcriptomic dataset (GSE116626) (16). Moreover, multiplex immunohistochemistry staining showed the adjacent position of Tfh cells and B cells *in situ*.

In this study, we investigated different subsets of CD4^+^T cells and found there was an increased proportion of Tfh cells in the peripheral blood of patients with IgAN, and the proportion of Tfh cells present was positively correlated with the severity of proteinuria. The Tfh cells were also observed to be hyper-functional. TNFSF14-TNFRSF14 interaction may participate in Tfh cells activating B cells in IgAN. We also demonstrated that Tfh and B cells were closely located in renal tissues of patients with IgAN and increased infiltration of CD4^+^T and B cells was positively correlated with the severity of renal injury. These findings highlighted the pathogenic role of follicular helper T cells in the development of IgAN and provided a potential target for therapeutic intervention.

## Materials and methods

### Sample collection

30 patients with primary IgA nephropathy diagnosed by renal tissue biopsy from the Department of Nephrology of Guangdong Provincial People’s Hospital (Guangzhou, China), and 29 age and gender matched healthy participants were recruited in this study. Exclusion criteria includes (1): Age < 18 years old (2); Less than 10 glomerulus in renal biopsy specimen (3); Secondary IgAN, such as Henoch-Schönlein purpura, lupus nephritis, Hepatitis B associated nephritis, cirrhosis associated nephritis etc. (4) Patients with serious diseases such as cardiovascular and cerebrovascular diseases, hematological disease, malignant tumors and other immune-mediated diseases (5); Patients who had reached ESRD. [eGFR < 15 (mL/min/1.73 m2), maintenance hemodialysis, peritoneal dialysis, kidney transplantation, etc.] (6); Patients with intestinal, upper respiratory, and urinary tract infections (7); Patients who have received any treatments including glucocorticoids, immunosuppressive drugs, antibiotics and renin-angiotensin-aldosterone system inhibitors.

Clinical information was collected, including gender, age and clinical test results such as serum creatinine, urinary protein creatinine ratio, 24 h urinary protein excretion, urea nitrogen, uric acid, total IgA, total IgG, serum protein, hemoglobin, triglyceride (TG), cholesterol (TC), C3, C4 and estimated glomerular filtration rate (eGFR). The eGFR was calculated using the Chronic Kidney Disease Epidemiology Collaboration (CKD-EPI) equation. The peripheral blood of all the patients was collected before any treatments and at the time of they received renal biopsy. The clinical indicators of patients were detected before treatments. The clinical characteristics were provided in **Table 1**. The MEST-C scores and LEE’s cores of all the included individuals were shown in **Table 2** and detailed histologic characteristics were given in **Table S1**.

**Table 1:** Clinic characteristics of patients.

Parameters	lgAN (n=30)
Age(Y)	35.7±9.1
Male/Female	17/13
BMI	22.43±3.46
eGFR(mL/min/1.73 m2)	80.03 ±29.15
CKD Stage 1	n=10
CKD Stage 2	n=13
CKD Stage 3	n=5
CKD Stage 4	n=2
Scr(umol/L)	106.47±59.42
Spro(g/L)	39.41 ±4.84
BUN(mmol/L)	6.01 ±2.24
24h Upro(g)	1.10±0.93
Upro:Ucr(mg/g)	1029.80±920.16
Uric acid(umol/L)	431.60±119.16
lgA(g/L)	3.17±0.95
lgG(g/L)	12.01 ±2.54
lgM(g/L)	1.34±0.65
C3(mg/L)	1214.55±217.69
C4(mg/L)	296.76±92.31
Hb(g/L)	124.83± 18.70
TC(mmol/L)	4.86±1.16
TG(mmol/L)	2.13±1.39

Values are means ± SD. eGFR, estimated glomerular filtration rate; CKD, chronic kidney disease; Scr, serum creatine; Spro, serum protein; BUN, urea nitrogen; 24 h Upro, 24 hours urinary protein quantitation; Upro:Ucr, Urinary protein creatinine ratio; Hb, hemoglobin; TC, cholesterol; TG, triglyceride.

**Table 2:** Pathological characteristics of patients.

	lgAN (n=30)
MEST-C classification	
Mesangial hypercellularity (M)	
M0	2 (6.7)
M1	28 (93.3)
Endocapillary hypercellularity (E)	
E0	28 (93.3)
E1	2 (6.7)
Segmental sclerosis (S)	
S0	11 (36.7)
S1	19 (63.3)
Tubular atrophy/interstitial fibrosis	
(T)	
T0	17 (56.7)
T1	9 (30)
T2	4 (13.3)
Crescents (C)	
C0	17 (56.7)
C1	9 (30)
C2	4 (13.3)
Lee's Classification	
I	0 (0)
II	8 (26.7)
III	8 (26.7)
IV	6 (20)
V	8 (26.7)

Values are n (%).

All subjects had been informed of the experiment content and signed informed consent when they were enrolled. 3 mL peripheral blood was extracted from each subject and collected in ethylenediaminetetraacetic acid (EDTA) anticoagulated tubes for subsequent experiments. This study was approved by the Ethics Committee of Guangdong Provincial People’s Hospital (KY-Q-2021-192-01, Guangzhou, China).

### Human peripheral blood mononuclear cells isolation

3 ml peripheral blood of patients with IgAN and healthy controls was collected in EDTA anticoagulated tubes. After centrifugation at 450 g for 5 min at room temperature (RT), the upper plasma was removed. Next, we added buffer solution (1× phosphate-buffered saline containing 0.2% bovine serum albumin) into the tube and diluted to 6 ml. The cell suspension was slowly added to the upper layer of 3 ml Ficoll solution (LymphoprepTM, STEMCELL Technologies, CAN) and density gradient centrifugation was performed (800 g × 20 min, up 6, down 2). The cells of the middle white membrane layer were collected and counted after they were treated with trypan blue stain.

### Flow cytometry

Single-cell suspensions were initially incubated with the human Fc receptor blocking reagent and stained for 20 min at 4°C using a cocktail of fluorochrome-conjugated antibodies. Antibodies included anti-CD45 BV650 (HI30), anti-CD3 Alexa Fluor 700 (OKT3), anti-CD4 BUV563 (SK3), anti-CD8a APC/Cy7 (HIT8a), anti-CD19 BV785 (HIB19), anti-CD56 BV711 (5.1H11), anti-CCR7 PE/Cy7 (GO43H7), anti-CD25 PE (M-A251), anti-PD-1 BV421 (EH12.2H7), anti-CCR4 BV605 (L291H4), anti-CCR6 PerCP/Cyanine5.5, anti-CXCR3 BV510 (G025H7), anti-CXCR5 PE/Dazzle 594 (J252D4), anti-CD127 BUV737 (HIL-7R-M21), anti-CD45RO FITC (UCHL1). All antibodies were purchased from BioLegend (San Diego, USA), except for anti-CD127 BUV737 (HIL-7R-M21, BD Biosciences, USA), anti-CD4 BUV563 (SK3, BD Biosciences, USA). Data were obtained using the BD LSRFortessa™ flow cytometer (BD Biosciences, USA) and analyzed using FlowJo software (BD Biosciences, USA).

### Single-cell RNA sequencing on peripheral blood mononuclear cells

PBMCs of 3 patients with IgAN and 4 healthy controls were isolated with cell viability exceeding 90% determined with trypan blue staining. An appropriate volume of cell suspension was calculated to contain ~1300 cells/ul. Single-cell capturing and library construction were performed using the Chromium Next GEM Single Cell 5’Reagent Kits v2 (10x Genomics, USA) according to the manufacturer’s instructions. The constructed libraries were sequenced on an Illumina NovaSeq platform to generate 2 × 150-bp paired-end reads. Cells from different samples were labelled using unique sample-specific oligonucleotides with DNA barcodes and then pooled for simultaneous library construction and sequencing. To assign sample identity to each cell based on a predefined barcode sequence.

### Singe-cell RNA sequencing data analysis

The CleanData were processed by the Cell Ranger pipeline (v3.0.1, 10x Genomics, USA), including demultiplexing, genome alignment (GRCh38), barcode counting and unique molecular identifier (UMI) counting. Doublet was removed by DoubleFinder (v2.0). Cells with more than 6,000 or less than 200 unique genes or having more than 25% mitochondrial genes were removed for quality control. We obtained a data matrix of a total of 18,125 cells (9,717 cells from patients with IgAN and 8,404 cells from healthy controls).

Seurat (v3.0.2) was used for further analysis including normalization, dimensional reduction, clustering and visualization. FindAllMarkers was used to identify and label these single cells. Each cell cluster was recognized on the basis of the markers validated and published in previous studies. The dimensionality reduction was performed using the RunTSNE function. According to the maker gene of CD4^+^T cell and B cell, we clustered 3701 CD4^+^T cells, including 1888 from patients with IgAN and 1813 from healthy controls, and 1264 B cells, including 591 from patients with IgAN and 673 from healthy controls. The identification of CD4^+^T cell subsets was based on the expression of characteristic genes of different CD4^+^T cells. These genes included *ICOS* and *CXCR5* for Tfh cells, *RORC* and *CCR6* for Th17 cells, *GATA3* and *CCR4* for Th2 cells, *TBX21* and *CXCR3* for Th1 cells, *FOXP3* and *IL2RA* for Treg, *SELL* and *CCR7* for naïve CD4^+^T cells. Subsets which didn’t have characteristic genes were defined as Others. The cell number and percentage for each CD4^+^T cell subsets was shown in **Table S2**

A supercell matrix of corresponding clusters was constructed to calculate the difference between cells in patients with IgAN and healthy controls by using LIMMA package (v3.44.3). Gene set variation analysis (GSVA) was performed using the GSVA package (v1.36.3). CellPhoneDB (v2.0) (https://github.com/Teichlab/cellphonedb) was used to analyze the intercellular communication networks and the intensity of receptor-ligand interaction between Tfh cells and B cells. The *P* values < 0.05 of ligand–receptor pairs were considered as significant. Statistical differences between patients with IgAN and health controls were based on the mean expression of receptor-ligands. The intercellular communication results including mean expression values and *P* values of receptor-ligands were shown in **Table S3**.

### Analysis of GEO IgAN kidney transcriptome dataset

A publicly available transcriptome microarray dataset of renal tissue from patients with IgAN was obtained from the GEO database (GSE116626). The associations among gene expression of *ICOS, MS4A1, PRDM1, AICDA, TNFSF14* and *TNFRSF14* were analyzed with the Spearman-test.

### Multiplex immunohistochemistry staining

Opal PolarisTM 7-Color Manual IHC Kit (NEL861001KT, Akoya Biosciences, USA) was used for multiplex immunohistochemistry (mIHC) staining. Formalin-fixed paraffin-embedded (FFPE) kidney tissues from patients with IgAN were dewaxed and rehydrated. After antigen retrieval, slides were blocked with antibody blocking (NEL861001KT) at RT for 30 min. The primary antibody, ICOS (1:200, 89601S, Cell Signaling Technology, Boston, USA) was incubated overnight at 4°C. Slides were washed and incubated with horseradish peroxidase-conjugated secondary antibody (NEL861001KT) at RT for 15 min. Finally, tyramide signal amplification (TSA) dye620 (1:100, NEL861001KT) was applied for 10 min after the wash. The second antibody, CD4 (1:500, Ab133616, Abcam, USA) was incubated at RT for 2 h and TSA dye520 (1:100, NEL861001KT) was then applied. The last antibody, CD20 (1:2000, Ab78237, Abcam, USA) was incubated at RT for 1h and TSA dye690 (1:100, NEL861001KT) was applied. Nuclei were stained with DAPI (C1006, Beyotime, Shanghai, China) at RT for 10 min. Slides were imaged and scanned using an Aokya Vectra PolarisTM Automated Quantitative Pathology Imaging System (Akoya Biosciences, USA), and multispectral images were acquired using Phenochart software, version 1.0.12 (Akoya Biosciences, USA) to unmix and remove autofluorescence. Slides were imported into HALO software version 3.2 (Indica Labs, CA, USA) for all subsequent steps, including annotation, training, and classification of multispectral slides.

### Statistical analysis

Data were presented as mean ± standard deviation (SD). Statistical differences were determined using non-parametric test (Mann-Whitney test). The Spearman-test was used for correlation analysis. All analyses were performed using the GraphPad PRISM software, version 8 (GraphPad Software, San Diego, CA, USA) and the R software, version 3.5.3. All *P* values were two-sided, and *P* < 0.05 was considered statistically significant.

## Results

### The proportion of Tfh, Th2, and Th17 cells was increased in patients with IgAN

**Figure 1:**
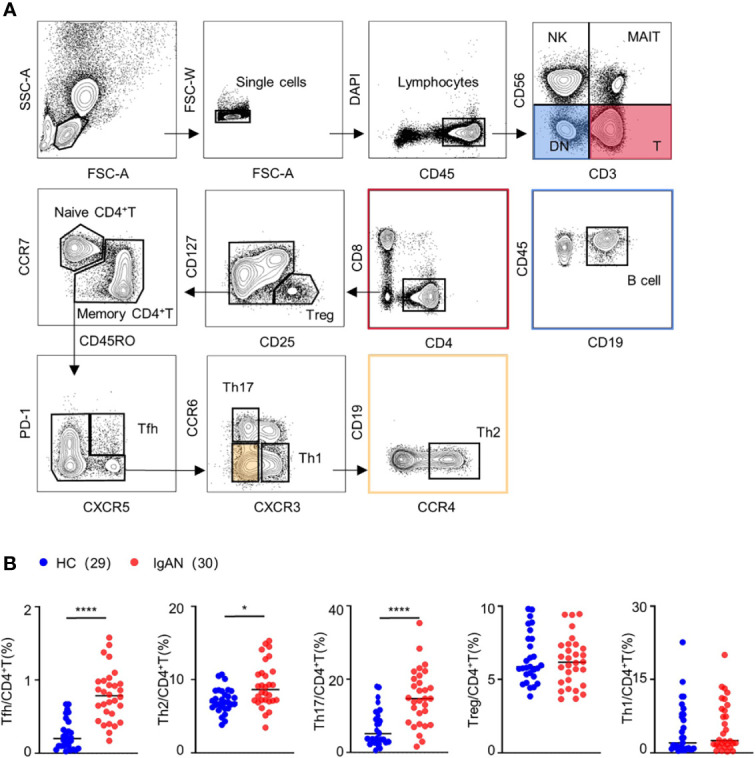
The proportion of Tfh, Th2, and Th17 cells was increased in patients with IgAN. **(A)** Gating strategy of CD4+T cell subsets in peripheral blood. **(B)** Percentage of CD4+T cell subsets, including Treg, Th1, Th2, Th17 and Tfh cell, in peripheral blood of patients with IgAN (n = 18) and healthy controls (HC, n = 17). Mann-Whitney test. *P < 0.05, ****P < 0.0001. Error bars represent SD.

In order to investigate the change of CD4^+^T cell subsets in patients with IgAN, we employed 18-colors flow cytometry to identify multiple CD4^+^T cell subsets (5) including naive CD4^+^T cell (CCR7^+^CD45RO^-^), Treg (CD25^+^CD127^-^), Th1 (CXCR3^+^), Th2 (CCR4^+^), Th17 (CCR6^+^), and Tfh cell (CXCR5^+^PD-1^+^) (**Figure 1A**). We collected the PBMCs from 30 patients with IgAN and 29 healthy controls for the experiment. The proportion of Tfh, Th2, and Th17 cells in patients with IgAN was significantly increased [For Tfh cells, 0.79 ± 0.40 (%) vs 0.14 ± 0.11 (%), *P* < 0.0001; Th2 cells, 9.74 ± 3.87 (%) vs 6.64 ± 1.36 (%), *P* = 0.0041; Th17 cells, 12.38 ± 7.87 (%) vs 3.42 ± 1.74 (%), *P* < 0.0001], compared with healthy controls. However, there was no significant difference in the proportion of Treg and Th1 cells between the two groups (**Figure 1B**). Therefore, Tfh, Th2, and Th17 cells might be involved in the pathogenesis of IgAN.

### Single-cell RNA sequencing identified CD4^+^T cells subsets in patients with IgAN

**Figure 2:**
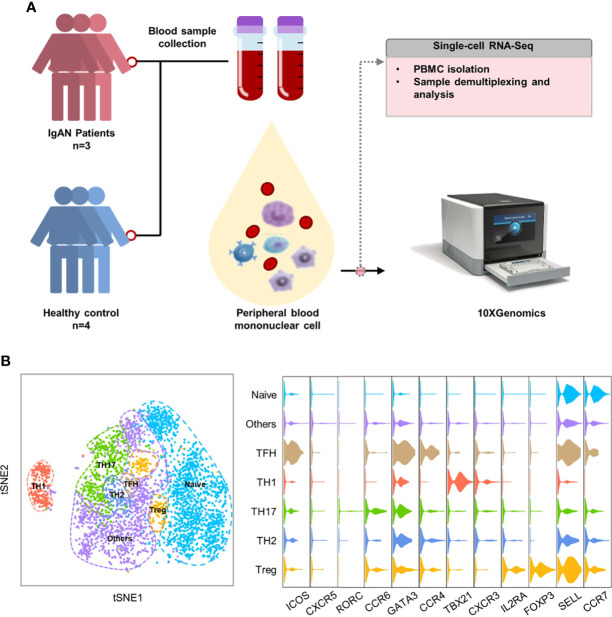
Single-cell RNA sequencing identified CD4^+^T cell subsets in patients with IgAN. **(A)** Single-cell RNA sequencing on PBMCs of patients with IgAN (n = 3) and healthy controls (n = 4). **(B)** tSNE dimensional reduction analysis of CD4^+^T cell based on single-cell transcriptomes. Each dot represents a single cell; dots in same colors indicate same cluster. Violin plot showing marker gene expression in each cluster. For naïve CD4^+^T cell (CCR7^+^SELL^+^), Th1 (TBX21^+^CXCR3^+^), Th2 (GATA3^+^CCR4^+^), Th17 (RORC^+^CCR6^+^), Tfh (CXCR5^+^ICOS^+^), Treg (IL2RA^+^FOXP3^+^) (t-SNE, t-distributed stochastic neighbor embedding).

To further understand the features of CD4^+^T cell subsets in patients with IgAN, we performed single-cell RNA sequencing on peripheral blood mononuclear cells from patients with IgAN (n = 3) and healthy controls (n = 4) (**Figure 2A**). Histopathological phenotypes of three patients with IgAN from the Department of Nephrology of Guangdong Provincial People’s Hospital were provided in the **Figure S1**. According to tSNE dimensionality reduction clustering results and their characteristic genes, we identified seven subgroups: naïve CD4^+^T cell (CCR7^+^SELL^+^), Th1 *(TBX21^+^CXCR3^+^*), Th2 (*GATA3^+^CCR4^+^*), Th17 (*RORC^+^CCR6^+^*), Tfh (*CXCR5^+^ICOS^+^*), Treg (*IL2RA^+^FOXP3^+^*) and others (**Figure 2B**).

### Tfh cells in IgAN manifested enhanced ability to support B cell response

**Figure 3:**
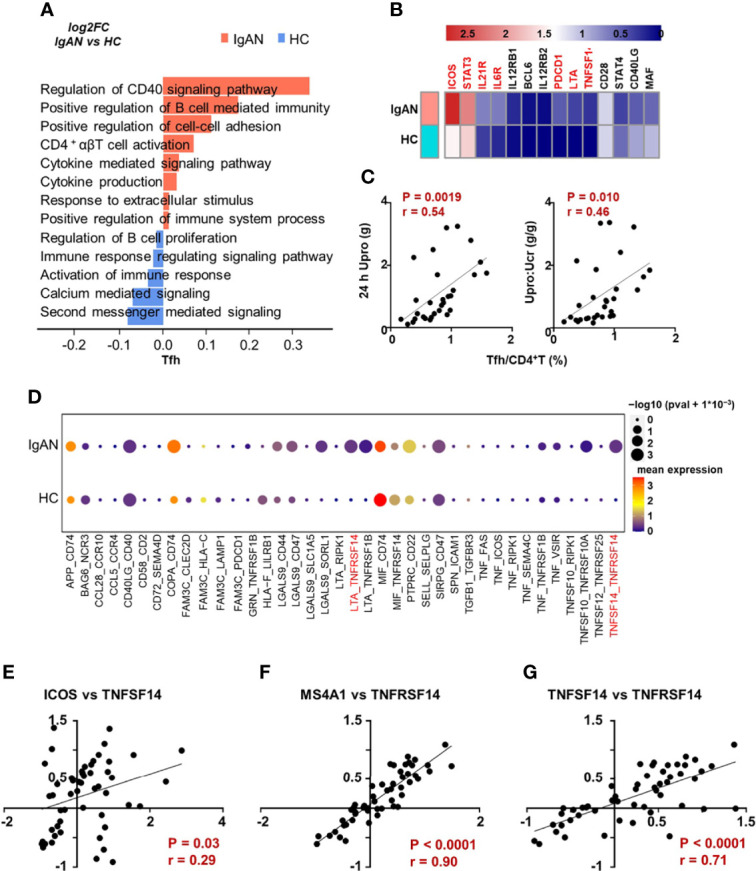
Tfh cells in IgAN manifested enhanced ability to support B cell response. **(A)** GSVA blot analysis of Tfh cells from patients with IgAN compared with HC based on single-cell transcriptomes. Red means upregulation while blue means downregulation. **(B)** The expression of several functional genes of Tfh cell in patients with IgAN and health controls. **(C)** Correlation analyses between the proportion of Tfh cells and clinical parameters in patients with IgAN (n = 30). **(D)** CellPhoneDB analysis between Tfh and B cells in patients with IgAN compared with HC based on single-cell transcriptomes. Color gradation represents mean expression. Circle represents *P* value. **(E–G)** Correlation analyses based on IgAN renal transcriptomic data (GSE116626). Spearman-test, *P* < 0.05. (GSVA, gene set variation analysis; 24 h Upro, 24 hours urinary protein quantitation; Upro : Ucr, Urinary protein creatinine ratio).

To further clarify whether the functional changes of Th cell participated in the pathogenesis of patients with IgAN, we analyzed signaling pathways of Th cell subsets based on the Gene Ontology (GO) database. The Th cells of patients with IgAN manifested a more active phenotype compared with healthy controls, since the genes were enriched in the pathways that promote inflammatory immune responses, such as cytokine secretion, antigen processing and presentation, and T cell chemotaxis (**Figure S2A**; **Figure 3A**). Notably, Tfh cells in patients with IgAN were enriched in pathways that regulate B cell differentiation and the CD40-CD40L signaling pathway. The cell adhesion and cytokine secretion pathways were also upregulated (**Figure 3A**). We also found that the expression of several functional genes of Tfh cell was increased in patients with IgAN compared with health controls, including *ICOS*, *STAT3*, *IL21R*, *IL6R*, *LTA* and *TNFSF14*, which might suggest the active phenotype of Tfh cells (**Figure 3B**).

Since the proportion of Tfh cells was significantly increased in patients with IgAN (**Figure 1B**), we analyzed the correlation between the proportion of Tfh cells and parameters for assessing kidney injury, including urinary protein creatinine ratio, 24 h urinary protein, and eGFR (CKD-EPI). We found that the proportion of Tfh cells was positively correlated with 24 h urinary protein and urinary protein creatinine ratio of patients with IgAN (**Figure 3C**), while the proportion of Th17 was negatively correlated with proteinuria (**Figure S2B**). However, the proportion of Th17 and Tfh cells was not correlated with eGFR (**Figure S2C**).

Considering the role of Tfh in B cell differentiation and development, we then investigated whether the phenotype of B cells was altered in patients with IgAN. We analyzed the transcriptomic data derived from single-cell RNA sequencing of B cells and found that the peripheral B cells of patients with IgAN showed enhanced differentiation and development from immature B cells towards mature B cells and germinal center B cells (**Figure S2D**). These results showed an active phenotype of B and Tfh cells in patients with IgAN and there was a potential association between them, suggesting that Tfh cells might activate B cell response and participate in the progression of IgAN.

To further clarify the underlying mechanism of interaction between Tfh and B cells in patients with IgAN, we analyzed the cell-cell communication network with the CellPhoneDB. Compared with healthy controls, the interaction of multiple receptor-ligands was stronger in patients with IgAN, especially, *LTA/TNFSF14* (LIGHT)-*TNFRSF14* (**Figure 3D**). The alteration of these signals between Tfh cells and B cells may be crucial for the development of IgAN. In order to verify these signals in the kidney of patients with IgAN, we analyzed the published renal transcriptomic data from patients with IgAN (GSE116626) (16)and found the expression of *ICOS*, marker of Tfh cell, was positively correlated with the expression of *TNFSF14* (**Figure 3E**). *MS4A1* (encoding CD20) was also positively correlated with *TNFRSF14* (**Figure 3F**). Moreover, the expression of *TNFSF14* was parallel to that of *TNFRSF14* in patients with IgAN, which was consistent with the results of CellphoneDB analysis derived from single-cell transcriptomic data (**Figures 3D–G**). In brief, Tfh cells may have a stronger ability to promote B cell response, and therefore exacerbate the progression of IgAN.

### Tfh cells co-localized with B cells in IgAN renal tissues

**Figure 4:**
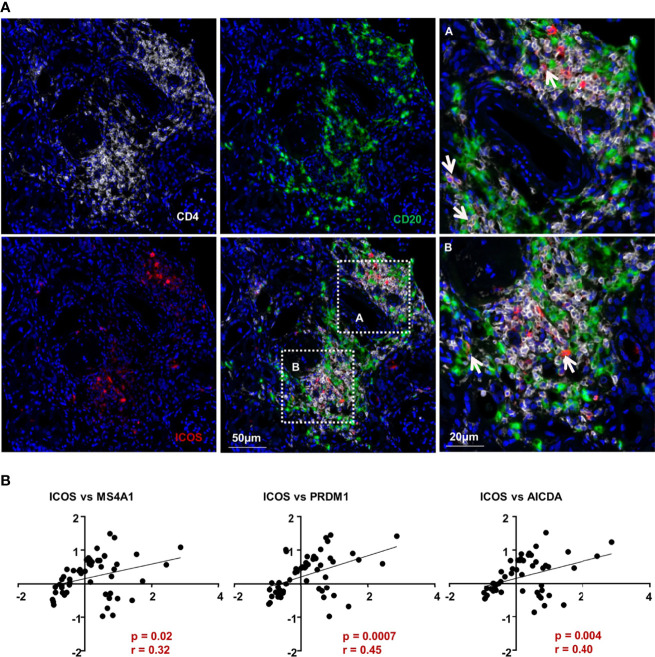
Tfh cells co-localized with B cells in IgAN renal tissues. **(A)** Multiplex immunohistochemistry staining on paraffin-embedded formalin-fixed renal biopsy tissues of patients with IgAN showing the co-location of Tfh cells and B cells (white arrow). **(B)** Correlation analyses of the Tfh and B cells based on renal transcriptomic database from patients with IgAN (GSE116626). (Spearman-test, *P < 0.05*).

As mentioned before, Tfh cells from PBMC of patients with IgAN showed a stronger tendency to promote B cell response. However, the interaction between Tfh cells and B cells *in situ* remained to be explored. Using multicolor fluorescence histochemical staining on renal biopsy tissues of patients with IgAN, we revealed that Tfh cells (CD4^+^ICOS^+^) were present in the kidney and mainly distributed around the glomeruli affected by nephritis. There were numerous B cells adjacent to Tfh cells, implicating possible direct contact between these cells (**Figure 4A**). These results suggest *in situ* interactions between B cells and Tfh cells. This was also verified by analysis of published renal transcriptome data (GSE116626), which shows the expression of ICOS was positively correlated with the expression of *MS4A1*. Meanwhile, the expression of *ICOS* was also positively correlated with that of *PRDM1* (encoding Blimp-1), which plays an essential role in the differentiation, maturation and antibody secretion of B cells (**Figure 4B**). The transcription of *AICDA (* 17), which regulates somatic hypermutations and class switching of B cells, was also positively correlated with that of *ICOS* (**Figure 4B**). In summary, our data suggests that Tfh cells may activate B cells *in situ* and contribute to renal impairment during the development of IgAN.

### CD4^+^T cell and B cell infiltration are associated with renal dysfunction in IgAN

**Figure 5:**
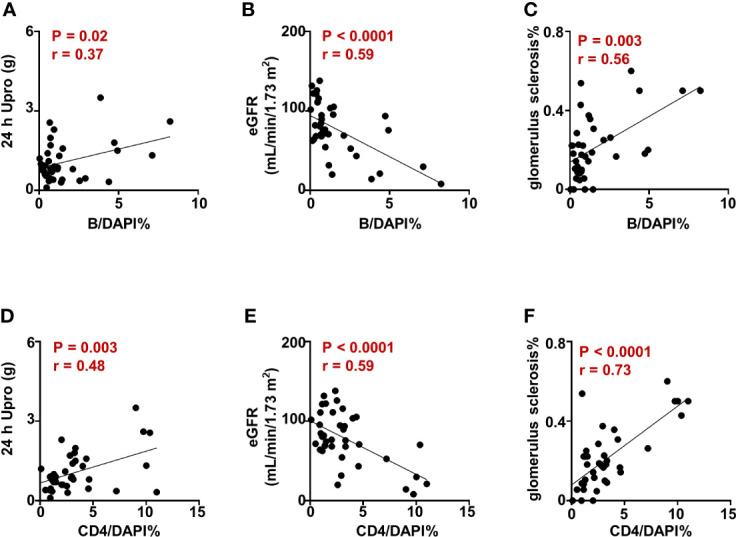
CD4^+^T cell and B cell infiltration are associated with renal dysfunction in IgAN. **(A–F)** Correlation analysis between the proportion of CD4^+^T and B cells in renal tissues and the parameters indicating renal impairment in patients with IgAN. Spearman-test, *P < 0.05*. (24 h Upro, 24 hours urinary protein quantitation; eGFR, estimated glomerular filtration rate).

To further clarify the infiltration of CD4^+^T cells and B cells in renal tissues, we performed multiplex immunohistochemistry staining on renal biopsy from patients with IgAN and analyzed the correlation between CD4^+^T cells and B cells infiltration and renal impairment parameters. We demonstrated that the ratio of infiltrated CD4^+^T cells and B cells was positively correlated with the severity of proteinuria (**Figures 5A**, **D**) and negatively correlated with eGFR in patients with IgAN (**Figures 5B**, **E**). Moreover, CD4^+^T cells and B cells infiltration was also positively correlated with the degree of glomerulus sclerosis (**Figures 5C**, **F**). Taken together, these results suggest that CD4^+^T and B cells invasion in renal tissues may play a crucial role in renal damage in IgAN.

## Discussion

A critical characteristic of IgA nephropathy (18–22) is immune complexes containing Gd-IgA1, which spotlight the pathogenic role of B cells. Understanding the characteristics and changes of CD4^+^T helper (CD4^+^Th) cells in IgAN is of great significance for elucidating the pathogenesis and development of the disease. Previous studies indicate that Tfh is involved in IgA and Gd-IgA1 productions *via* secretion of IL-21, which promotes the increase of activation-induced cytidine deaminase and the occurrence of B cell somatic hypermutation in patients with IgAN (23, 24).

In the current study, we report that peripheral Tfh was hyper-functional in regulating B cell response-related pathways, such as the CD40-CD40L signaling pathway, cell adhesion and cytokine secretion pathways, and positively correlated with the proteinuria level in patients. Furthermore, the infiltration of CD4^+^T and B cells was increased in the kidney of patients with IgAN, which may contribute to the damage of the kidney. Interestingly, the infiltrated CD4^+^T cells were adjacent to B cells, especially Tfh cells. According to the correlation analysis between *ICOS* and *PRDM1/AICDA*, *TNFSF14* and *TNFRSF14*, renal transcription data also suggests that Tfh cells in patients with IgAN have strong interaction with B cells. Correspondingly, B cells exhibit higher ability of differentiation and activation in IgAN. We also found that the expression of several functional genes of Tfh cell such as *ICOS*, *STAT3*, *IL21R* and *IL6R* was increased in patients with IgAN compared with health controls. Combined with all these results, it suggests that the interaction between Tfh and B cells may participate in the progression of IgAN progression.

The tumor necrosis factor superfamily of ligand (TNFSF) and its receptor (TNFRSF) is essential for communication between immune cells, and its dysfunction can lead to the development of autoimmune diseases (14). TNFSF14 (LIGHT), predominantly expressed in activated immune cells, has a wide range of roles in immune system, such as being a key signal in secondary lymphoid organs development and TLOs formation (25–27). Meanwhile, LIGHT-mediated intestinal inflammation not only stimulates excessive production of IgA in the intestine, but also leads to a defect in IgA transport in the intestinal lumen (15). Therefore, enhanced interaction of TNFSF14-TNFRSF14 between Tfh and B cells in IgAN indicates a potential pathogenic role of LIGHT in IgAN.

Renal damage can expose the antigenic target, which can be recognized and processed by antigen-presenting cells in local microenvironment (28). The interaction between Tfh cells and B cells in renal cells can also organize structures that comprise functional germinal centers, defined as tertiary lymphoid organ (TLOs) (8, 29, 30). Past research has found TLOs in various CKD (31–33), and TLOs were observed in 37.5% of patients with IgAN (34). Interestingly, we found that Tfh cells co-localized with B cells on renal biopsy tissues of patients with IgAN, suggesting the existence of TLO in IgAN. Further, our research shows that some of the infiltrated B cells were IgG^+^IRF4^+^ in kidney of patients with IgAN (data not showed), cluing that the pathogenic mechanism of TLO.

There are several obvious limitations in our research. Although we describe the alterations of Tfh cells in several aspects in patients with IgAN, the potential disease-specific costimulatory signals between Tfh and B cells discovered in our study need to be verified by further studies. The detailed mechanism of kidney injury caused by Tfh-B cells is not clear yet. More *in vivo* and *in vitro* experiments need to be designed for further study, to provide more specific targets for advancing the treatment of IgAN.

In conclusion, we demonstrated the importance of Tfh cells in activating B cell response during the progression of IgAN and the potential relationships between them. Interfering with the interaction between Tfh and B cells may be a promising strategy for developing novel therapies for IgAN.

## Data availability statement

The data that support the findings of this study have been deposited into CNGB Sequence Archive (CNSA) (35) of China National GeneBank DataBase (CNGBdb) (36) with accession number CNP0002798.

## Ethics statement

The studies involving human participants were reviewed and approved by Ethics Committee of Guangdong Provincial People’s Hospital. The patients/participants provided their written informed consent to participate in this study.

## Author contributions

XQY, Z-XL, C-YG, MS designed the research. WSD, C-YG, XY and MTF performed the experiments. WSD and XY analyzed the data. WSD wrote the manuscript, which was revised by XQY, Z-XL, C-YG, MJS, LL, Z-BZ and ZMY. All authors have read and approved the final manuscript. All authors contributed to the article and approved the submitted version.

## Funding

This work was supported by grants from National Natural Science Foundation of China (81920108008, 82000725) and Guangdong-Hong Kong-Macao-Joint Labs Program from Guangdong Science and Technology (2019B121205005).

## Conflict of interest

The authors declare that the research was conducted in the absence of any commercial or financial relationships that could be construed as a potential conflict of interest.

## Publisher’s note

All claims expressed in this article are solely those of the authors and do not necessarily represent those of their affiliated organizations, or those of the publisher, the editors and the reviewers. Any product that may be evaluated in this article, or claim that may be made by its manufacturer, is not guaranteed or endorsed by the publisher.

## Supplementary material

Supplementary Figure 1 Histopathological phenotypes in 3 IgAN patients with single-cell RNA sequencing. Immunofluorescent staining of IgA, electron microscope image and PASM staining of kidney biopsy from subjects with single-cell RNA sequencing.

Supplementary Figure 2 Single-cell RNA sequencing analysis of Th cells and B cells in patients with IgAN. **(A)** GSVA blot analyses of Th1, Th2, Treg and Th17 cells from patients with IgAN compared with HC based on single-cell transcriptomes. Red means upregulation while blue means downregulation. **(B, C)** Correlation analysis between the proportion of Th17 and Tfh cells measured by flow cytometry in patients with IgAN and clinical parameters (n = 30). **(B)** The ratio of Th17 and proteinuria (Spearman-test, P < 0.05). **(C)** The proportion of Th17 and Tfh cells and eGFR (Spearman-test, P > 0.05). **(D)** GSVA blot analysis of B cells from patients with IgAN compared with HC based on single-cell transcriptomes. Red means upregulation while blue means downregulation. (24 h Upro, 24 hours urinary protein quantitation; Upro : Ucr, Urinary protein creatinine ratio; eGFR, estimated glomerular filtration rate).

Supplementary Table 1 Detailed histopathological characteristics of 30 patients with IgAN.

Supplementary Table 2 Information of CD4^+^T based on single-cell RNA sequencing.

Supplementary Table 3 The results of intercellular communication analysis between Tfh and B cells.
