# Supplementary figures and images for: Increased proportion of follicular helper T cells is associated with B cell activation and disease severity in IgA nephropathy
Source: Front Immunol. 2022 Aug 2;13:901465. doi: 10.3389/fimmu.2022.901465 (PMC9381139; doi:10.3389/fimmu.2022.901465)

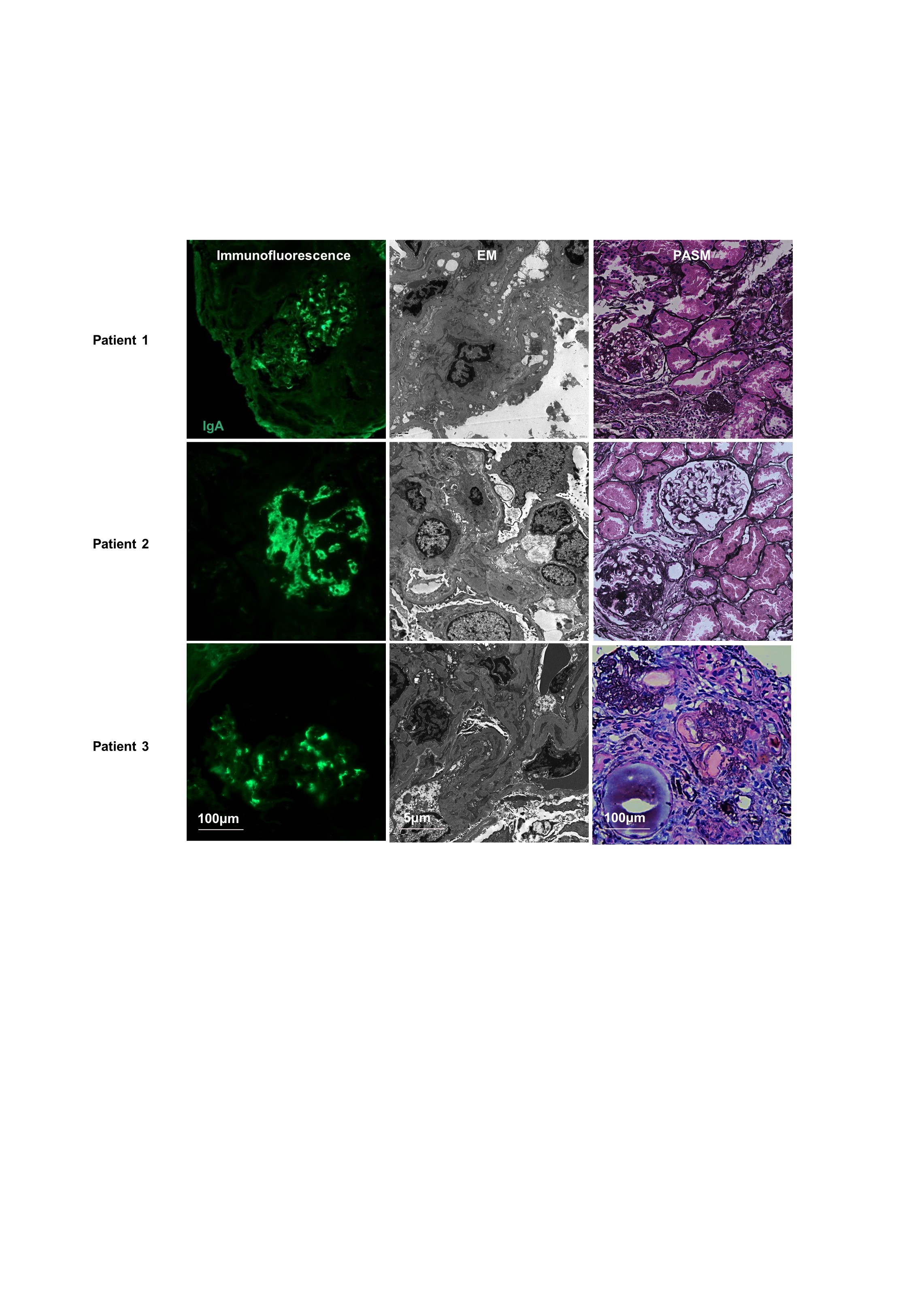

Supplement: Supplementary Figure 1 — Histopathological phenotypes in 3 IgAN patients with single-cell RNA sequencing. Immunofluorescent staining of IgA, electron microscope image and PASM staining of kidney biopsy from subjects with single-cell RNA sequencing. [file Image_1.jpg]

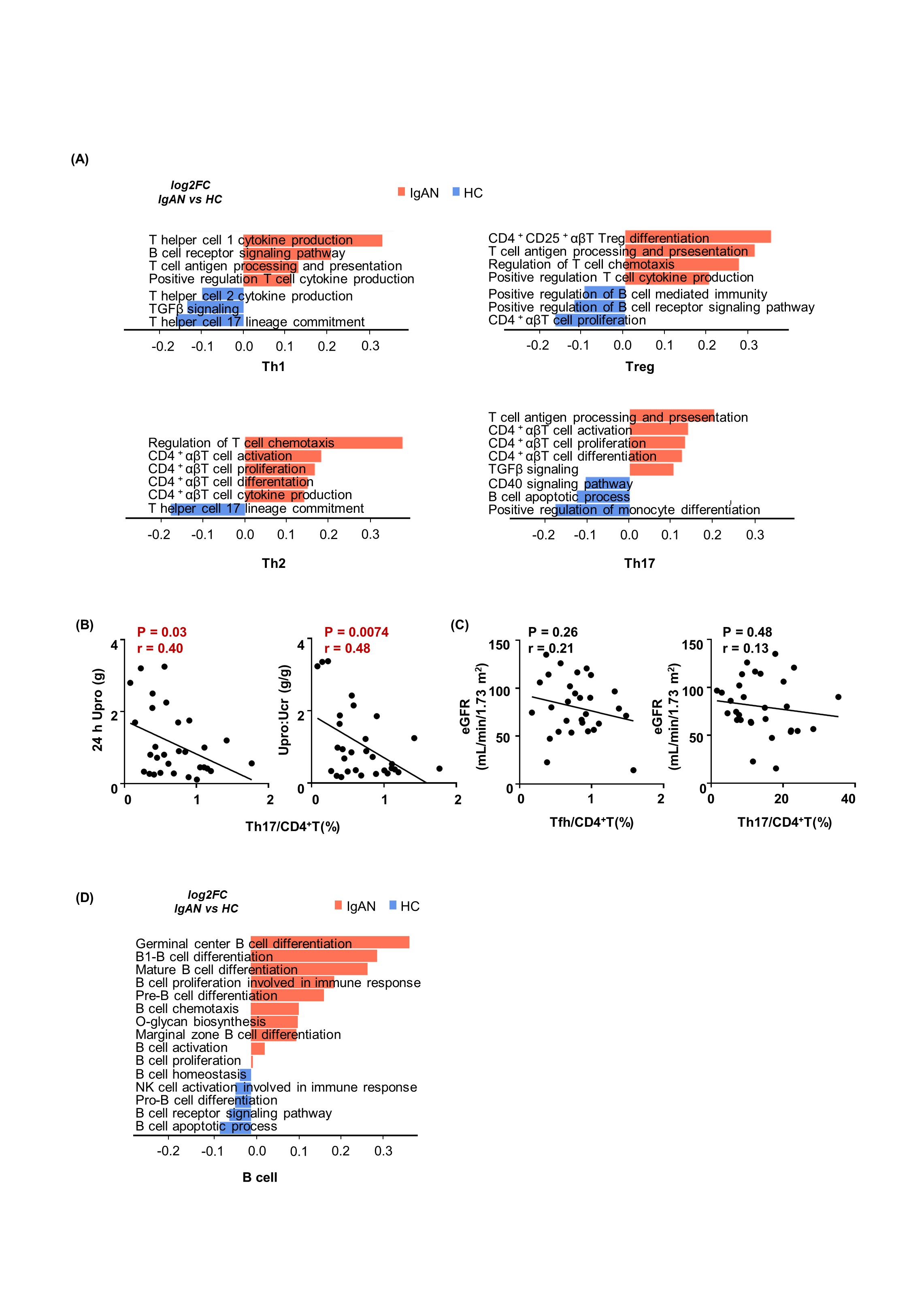

Supplement: Supplementary Figure 2 — Single-cell RNA sequencing analysis of Th cells and B cells in patients with IgAN. (A) GSVA blot analyses of Th1, Th2, Treg and Th17 cells from patients with IgAN compared with HC based on single-cell transcriptomes. Red means upregulation while blue means downregulation. (B, C) Correlation analysis between the proportion of Th17 and Tfh cells measured by flow cytometry in patients with IgAN and clinical parameters (n = 30). (B) The ratio of Th17 and proteinuria (Spearman-test, P < 0.05). (C) The proportion of Th17 and Tfh cells and eGFR (Spearman-test, P > 0.05). (D) GSVA blot analysis of B cells from patients with IgAN compared with HC based on single-cell transcriptomes. Red means upregulation while blue means downregulation. (24 h Upro, 24 hours urinary protein quantitation; Upro : Ucr, Urinary protein creatinine ratio; eGFR, estimated glomerular filtration rate). [file Image_2.jpg]

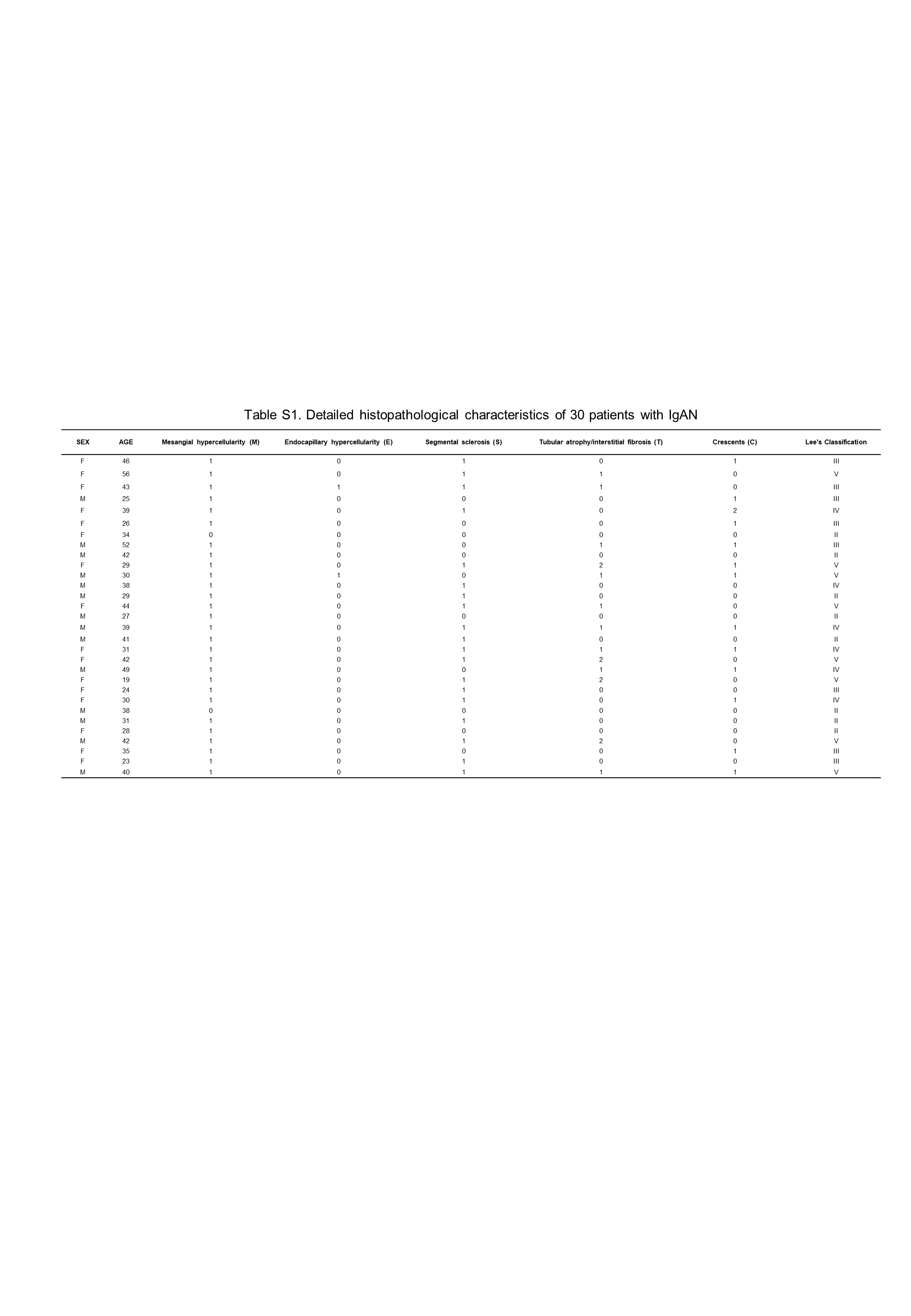

Supplement: Supplementary file 4 [file Image_3.jpeg]

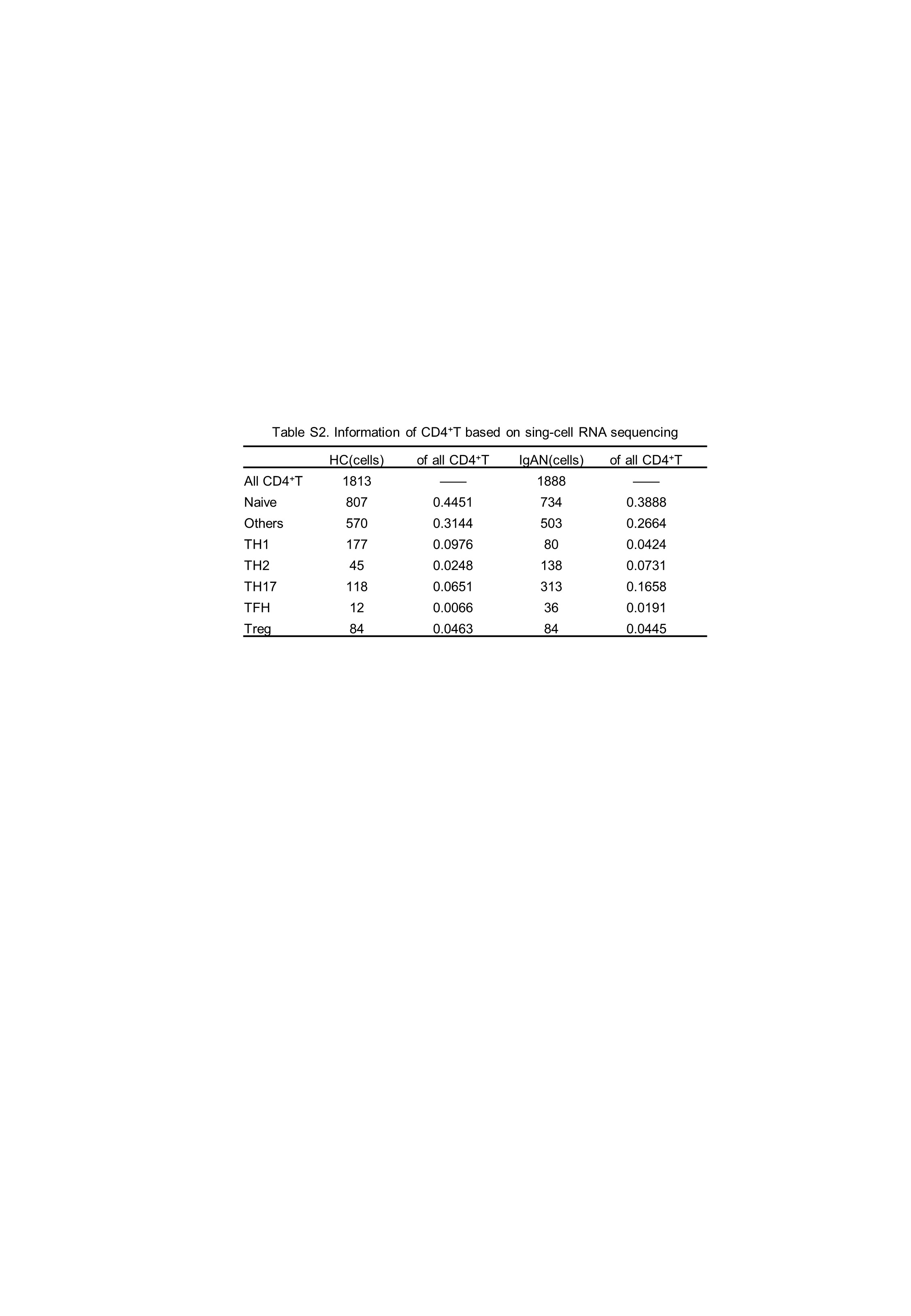

Supplement: Supplementary file 5 [file Image_4.jpeg]
